# Latest Research Trends in Fall Detection and Prevention Using Machine Learning: A Systematic Review

**DOI:** 10.3390/s21155134

**Published:** 2021-07-29

**Authors:** Sara Usmani, Abdul Saboor, Muhammad Haris, Muneeb A. Khan, Heemin Park

**Affiliations:** 1 School of Electrical Engineering and Computer Science (SEECS), National University of Sciences and Technology (NUST), Islamabad 44000, Pakistan; susmani.msit15seecs@seecs.edu.pk (S.U.); mharis.msit15seecs@seecs.edu.pk (M.H.); 2 Department of Electrical Engineering (ESAT), Katholieke Universiteit (KU) Leuven, 3000 Leuven, Belgium; abdul.saboor@esat.kuleuven.be; 3 Department of Software, Sangmyung University, Cheonan 31066, Korea; muneebkhan046@gmail.com

**Keywords:** fall detection, fall prevention, machine learning, review paper

## Abstract

Falls are unusual actions that cause a significant health risk among older people. The growing percentage of people of old age requires urgent development of fall detection and prevention systems. The emerging technology focuses on developing such systems to improve quality of life, especially for the elderly. A fall prevention system tries to predict and reduce the risk of falls. In contrast, a fall detection system observes the fall and generates a help notification to minimize the consequences of falls. A plethora of technical and review papers exist in the literature with a primary focus on fall detection. Similarly, several studies are relatively old, with a focus on wearables only, and use statistical and threshold-based approaches with a high false alarm rate. Therefore, this paper presents the latest research trends in fall detection and prevention systems using Machine Learning (ML) algorithms. It uses recent studies and analyzes datasets, age groups, ML algorithms, sensors, and location. Additionally, it provides a detailed discussion of the current trends of fall detection and prevention systems with possible future directions. This overview can help researchers understand the current systems and propose new methodologies by improving the highlighted issues.

## 1. Introduction

Aging is a worldwide problem related to life expectancy [1]. The World Health Organization (WHO) states that the elderly population is 20% of the world’s population [2]. Another report states that older people (above 65 years) will increase to 1.5 billion by the end of 2050 [3]. In general, old age reduces the overall physical, cognitive, and sensory functionalities [4,5]. Therefore, an older adult faces difficulty performing routine tasks such as walking, jogging, eating, and dressing up [6,7,8]. Falling is a significant challenge in the elderly group that can reduce life expectancy. Approximately 35% of people (above 65 years) have one or more falls per year [9]. In addition to old age, several other factors such as environment, physical activity, and cardiovascular disorders cause falls. It is a major source of physical injuries, and often, these injuries require hospitalization [10,11,12]. Annually, 37.3 million falls need medical attention, and 0.65 million falls resulting in deaths [13].

A fall can be described as an unpredicted event leading the participants to rest on the lower level (ground or floor) [14]. As a result, it causes injuries that can often be fatal [15,16]. Psychological grievances are also considered as the consequence of falls. People may suffer from anxiety, depression, activity restriction, and fear of falling [17,18]. The primary physiological issue in older adults is fear of falling, restricting their Activities of Daily Life (ADL) [19]. This fear leads to activity restriction, which may lead to inadequate gait balance and weakened muscle that affects the mobility and independence of older adults. Therefore, remote/wearable technologies are required to track, detect, and prevent falls for improving the overall quality of life (QoL) [20,21]. For this purpose, understanding of falls can be classified as fall prevention and fall detection. Fall detection refers to the detection of a fall using sensors/cameras to summon help. In contrast, fall prevention aims to avert falls by observing human locomotion. Numerous systems have been developed using different sensors and algorithms to detect and prevent the fall.

The authors of [14,22] presented an overview of the fall detection techniques. However, both the studies include relatively old literature published in 2007 and 2008, respectively. Mubashir et al. [23] classified the fall detection approaches into wearable, ambient, and camera-based approaches. Similarly, Igual et al. [24] talks about the issues and trends in fall detection schemes. The study [25] is specific to fall detection using wearable sensors. All the above-mentioned reviews only discuss fall detection schemes with no interest in fall prevention. In 2014, Delahoz et al. [26] presented a review on fall detection and prevention techniques. Recently, Saboor et al. [27] published a review on gait analysis using machine learning. However, only 14% of their studies are specific to fall detection and prevention. Ren et al. [28] present a comprehensive overview of fall detection and prevention techniques. However, most presented schemes use statistical approaches that often generate many false alarms during detection and classification. Furthermore, statistical approaches are less efficient in the presence of complex and nonlinear problems [29]. In general, gait analysis for fall detection and prevention often generates noisy data during the acquisition. Statistical methods are generally sensitive to noisy data that leads to performance degradation [30]. Therefore, the latest research incorporates Machine Learning (ML) because of high classification accuracy for fall detection and prevention. Recently, Islam et al. [31] presents a review on fall detection using deep learning techniques. However, the scope of the review is limited to deep learning techniques for fall detection only. This paper aims to provide an overview of studies using ML for fall detection and prevention. The overall contributions of this paper are as follows:

- It provides an overview of the fall detection and prevention systems using wearables and non-wearables.
- It elaborates on the frequently used ML algorithms in fall detection and prevention.
- It provides a detailed analysis of the recent state-of-the-art studies. The analysis covers the dataset, participants, ML algorithms, acquisition sensors, and their placements.
- It evaluates performance parameters such as accuracy, sensitivity, and specificity for different combinations of ML algorithms, sensors, and placements.
- It provides a detailed discussion on the latest trends in fall detection and prevention systems along with the future directions.

The paper’s overall structure is as follows: Section 2 provides an overview of fall detection and fall prevention approaches. Section 3 elaborates on the frequently used ML techniques in fall detection and prevention. Section 4 is the proposed methodology, followed by the discussion and analysis in Section 5. Section 6 consists of discussion and future work. Finally, Section 7 concludes the work.

## 2. Fall Detection and Prevention Systems

The development of fall detection and prevention systems has become a hot research topic during the last few years. Various approaches are used for developing such systems. These systems are classified into two broader categories: wearable systems and non-wearable systems.

### 2.1. Non-Wearable Systems

Non-wearable systems are composed of sensors placed around the human proximity for data/gait monitoring. These systems are further subdivided into vision-based sensors, and floor-based sensors [32]. Vision sensors such as cameras, infrared sensors, and Laser Range Scanners (LRS) [33] take optical measurements and use image processing for analysis. Video surveillance is a common type of such system, which captures images and uses different algorithms to determine fall occurrence. In contrast, floor-based sensors such as Ground Reaction Force (GRF) sensors and pressure sensors observe the force extracted by human feet to observe the fall [34]. The number of sensors varies from experiment to experiment. The primary drawback of non-wearable systems is their limited coverage. Such systems can be implemented at offices, homes, and experiment labs, making them less scalable and expensive. Non-wearable systems also compromise users’ privacy [35,36]. Therefore, it is not optimal to use such systems for most real-life applications.

### 2.2. Wearable Systems

**Figure 1:**
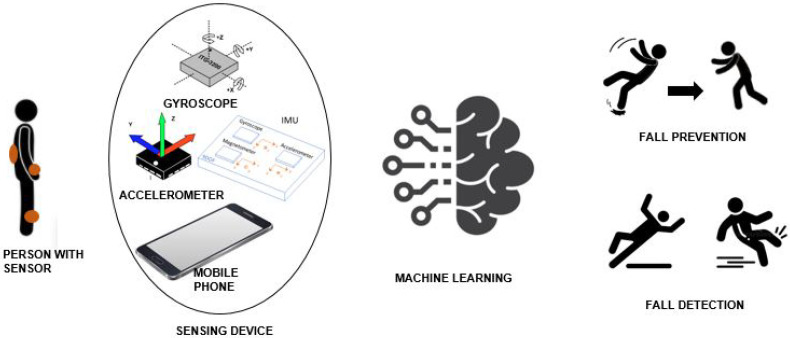
An Overview of Fall Detection and Prevention System.

Wearable systems consist of devices/sensors that can be attached to the human body for data collection. Wearable systems consist of accelerometers, gyroscopes, magnetometers, IMUs, etc. [37]. An overview of wearable sensors is given in Table 1. The primary advantage of wearable systems is their ability to collect data outside the laboratory environment [38]. Therefore, such systems are feasible for analyzing fall detection or for preventing falls while performing ADLs. These sensors are often embedded in smartphones that can collect data without investing in any new equipment [39]. Additionally, they provide better privacy than non-wearable systems. However, wearable devices have limited lifetime processing power [40,41]. Furthermore, the wearable’s data need further processing using statistical or ML algorithms for decision making, as shown in Figure 1.

The statistical approaches often lead to low classification accuracy and prove to be less efficient with noisy data. Therefore, ML techniques are widely used for fall identification and prevention [43]. Basic fall activities that are identified are falling forward, falling backward, falling sideways, spinning clockwise, and spinning anticlockwise [44]. The ML algorithms classify fall activities from non-fall activities based on the classifier being trained [44,45,46]. Similarly, these ML classifiers identify the abnormalities in gait and try to prevent falls using techniques such as muscle stimulation [47]. An overview of a general system is presented in the next subsection.

### 2.3. System Overview

**Figure 2:**
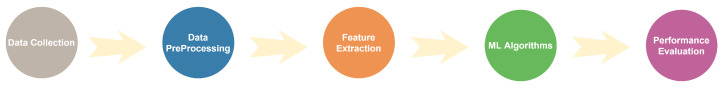
Procedure for fall detection and prevention.

The overall system for fall detection and prevention consists of the five steps as shown in Figure 2. The first step is data collection depending on the application requirements. There are various data collection methods, i.e., public datasets, controlled environments, and realistic environments. Publicly available datasets include gait features that can be used to develop such systems [48,49]. In contrast, a lab or realistic environment uses wearable [50,51,52,53] or non-wearable devices [54] for data acquisition.

In general, the acquired data are noisy. Therefore, preprocessing helps to remove the noisy and unwanted signals from the data. For that, the system uses preprocessing filters such as Kalman Filter [55] and Median Filter [56] etc. The third step is feature extraction to obtain the desired features from the preprocessed data. The features can vary from experiment to experiment performed by different researchers. For example, in speech recognition, the desired features are sound length, noise ratio, matching filters, and relative power. Similarly, edges and objects are used as desired features in computer vision applications. In contrast, fall detection or prevention applications require a change in acceleration, rotation, or angular velocity as the desired feature set. The slight change in any of these parameters helps visualize the gait changes, resulting in fall detection or fall prevention. Therefore, the mean, standard deviation, and variance of these features are considered valuable data for such application. Overall, feature selection is a crucial step, as classification accuracy heavily relies on the selected features. Feature selection also reduces the dataset volume and cost of the pattern recognition process. Features can be selected using filter methods or wrapper methods [57,58].

A large number of features can cause overfitting, while fewer features may cause underfitting. Therefore, this step requires additional attention to enhance the overall performance of the system. The fourth step uses ML algorithms to classify irregular gait, falls, or ADL. Generally, it divides the data into training and testing data. The ratio of each data type depends on the experiment of system design. This step applies the ML algorithms on training data to identify fall activities or irregular gait for fall prevention. After training the classifier, it uses test data for the performance evaluation. This step includes various matrices such as the accuracy, sensitivity, and specificity of the results obtained to analyze the system’s overall performance. As we can see, the ML algorithms help in identifying fall detection or prevention. Therefore, the next section discusses the functionality of major machine learning algorithms used for fall detection and prevention.

## 3. Machine Learning Algorithms

ML provides a learning ability to the system based on the dataset and trends in data. During the data collection process, sensors provide the data associated with different fall parameters. Thus, the ML algorithms are used to process the data to classify or identify fall activities based on the application requirements. The most widely used ML algorithms for fall detection and prevention are the following.

### 3.1. Support Vector Machine (SVM)

The SVM is a supervised machine learning model to find a hyperplane in an n-dimensional space (where N is the number of features that distinctly divide the data). In general, a hyperplane reduces an n-dimensional space to (n-1)-dimensional space. The SVM can be used for classification and regression, but it is mainly used for classification problems [59]. There are two types of SVM: linear and non-linear. The linear classifier [60] assumes that each data point is isolated linearly. Therefore, it separates the two classes by identifying the optimal hyperplane with maximizing the margin. However, in the widely used non-linear classifier [61], the data are first mapped with a kernel, and a discriminant function is found. That discriminant function is associated with the hyperplane in the transformed space. In addition, the kernel is used in various machine learning algorithms for pattern analysis.

**Figure 3:**
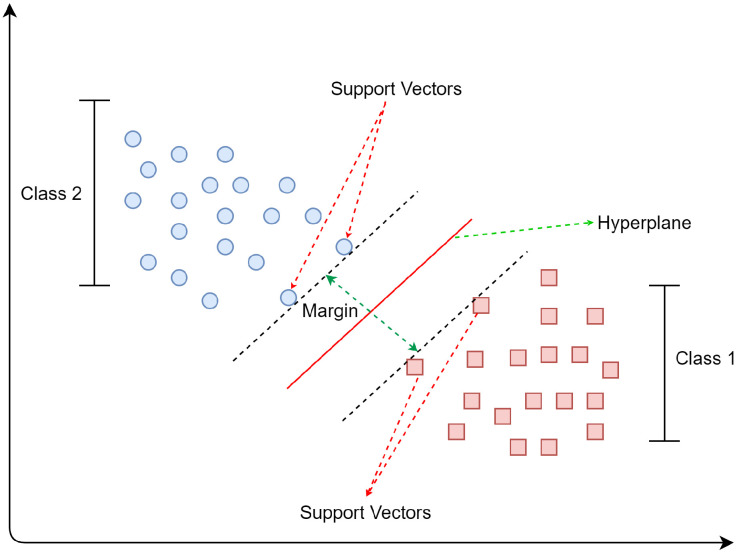
Support vector machine algorithm.

In SVM, the data points are available in a plane that can be obtained from different classes based on their locations. A hyperplane [62] acts as a decision boundary to classify these data points. There are multiple hyperplanes available, but the main objective is to find the plane that has the maximum distance between the support vectors, as shown in Figure 3. Support vectors are the data points that lie near the hyperplane and play an important role in identifying the position of the hyperplane. The SVM tries to generate a decision boundary [63] where the gap between the classes is the most comprehensive. Hence, it is a widely used classifier for differentiating fall activities from daily life activities. For example, class 1 in Figure 3 represents the regular walking pattern and class 2 shows the fall feature.

### 3.2. Artificial Neural Network (ANN)

**Figure 4:**
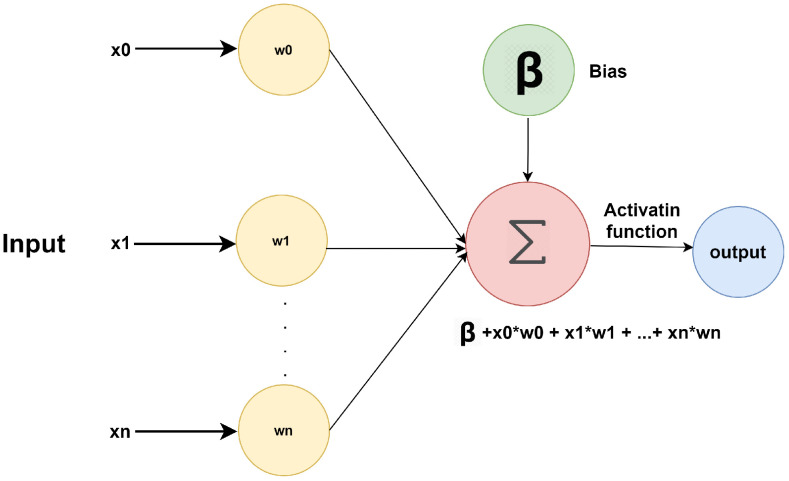
Artificial neural network algorithm.

An ANN is a machine learning algorithm whose methodology is inspired by the working of the human brain [64]. The human brain is composed of billions of neurons that process information in the form of electric signals. The neurons generally make a decision based on the electric signal strength. Similarly, the ANN consists of many interconnected processing elements to solve a specific problem, as presented in Figure 4.

Here, x variables (x0, x1, x2, …, xn) are the input to the ANN, and w variables (w0, w1, w2, ……, wn) are the weights of each input. Each weight represents the strength of the input signal. The bias function (b) shifts the activation function up or down according to needs [65]. The activation function translates the input function to the ANN output signal. Along with the input and output layer, there can be various hidden layers between these two layers to classify data better. Therefore, ANN is called a Multi-Layer Perceptron (MLP) [66]. In the presence of a hidden layer, the ANN output signal of one layer can be used as input for the next layer. The ANN requires a trainer for the identification of the response for every given input. Therefore, additional training is required to predict the output for every input. A cost function is used to find the difference between the actual value and the predicted value [67]. The cost function is calculated for every layer in the network, and weights are adjusted for the following input. This process is repeated until the minimized cost function is obtained, providing the minimum difference between the actual value and predicted value. Therefore, it can help predict or prevent falls.

### 3.3. Random Forest (RF)

**Figure 5:**
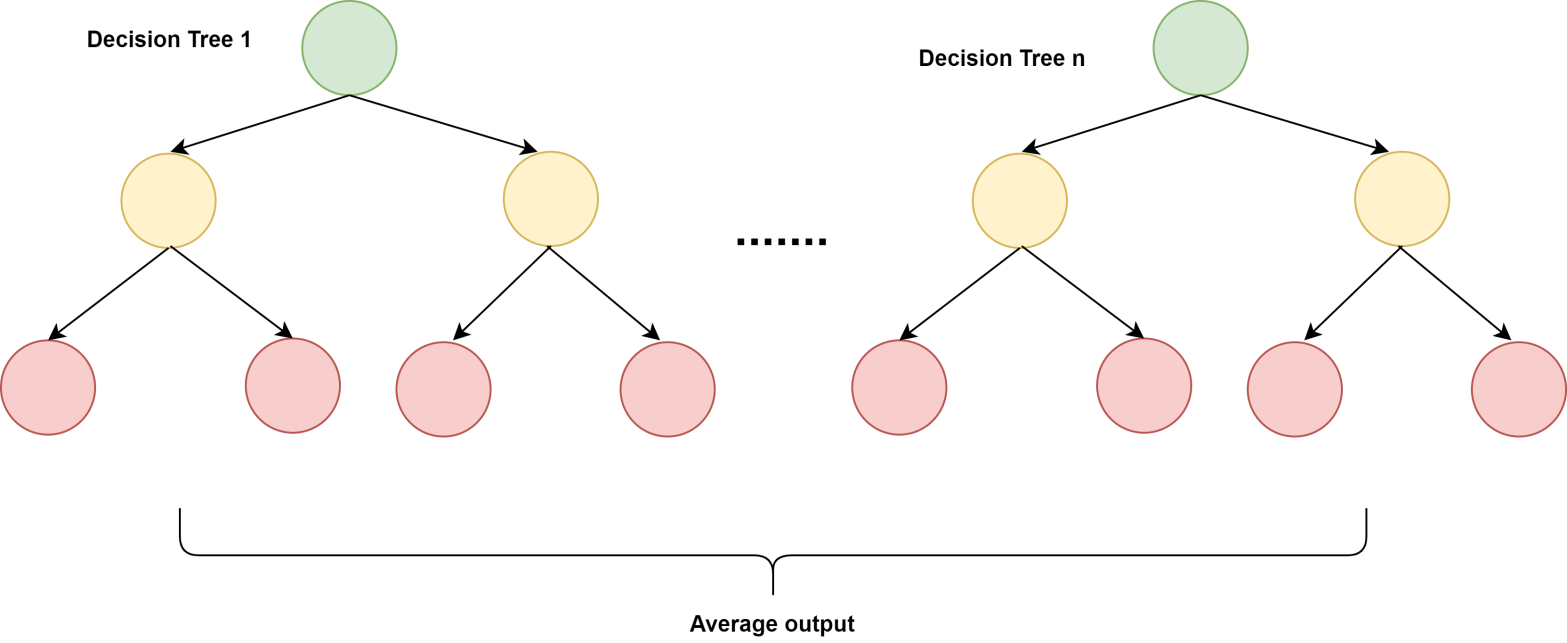
Random Forest Algorithm.

Random forest is also a supervised classification algorithm consisting of a large number of decision trees, as shown in Figure 5. A decision tree consists of nodes (representing a test) and branches that show the specific test outcome [68]. After that, it builds the classification based on the paths in the tree. However, the decision tree is sensitive to training data. Small changes in training data cause significant changes in the overall tree structure. Therefore, bagging is used in the random forest. In bagging, each tree randomly selects a random sample from the dataset to generate multiple decision trees [69]. Every tree in the random forest forms a class, and voting is performed to identify the best prediction class of the model.

Random forest follows the wisdom of crowds rule. Every tree applies the decision tree model to predict the result. The class with the majority of the votes (i.e., predicted by multiple trees in the random forest) is considered the predicted class [70]. Therefore, a random forest can be used to model categorical data to avoid the overfitting problem. Hence, it is helpful for the classification of ADL from fall activities.

### 3.4. k-Nearest Neighbors (kNN)

kNN is a supervised learning algorithm for solving classification and regression models. kNN assumes that similar things lie in close proximity [71]. Hence, it tries to find the distance between the data points known as the Euclidean distance. Initially, a k value is selected to choose the number of neighbors for a data point. The k value is responsible for marking the boundaries of clusters or classes [72]. kNN generates a sorted list containing the distance between each point and the other points. Eventually, the first k entries are selected for regression and classification. kNN is very simple and computationally inexpensive and does not require additional assumptions [73]. Therefore, it is widely used in recommendation systems [74]. Therefore, kNN can help distinguishing fall behaviors from non-fall behaviors.

### 3.5. k-Means

k-means is a simple and popular unsupervised machine learning algorithm. k-means groups similar data points together for discovering underlying patterns [75]. It identifies the various clusters (k) in a dataset for classification [76]. A cluster consists of similar data points that are specific to activities such as gait of fall. By defining k, it determines the number of centroids needed in the dataset [77]. The centroid is the center of each cluster. Each data point is associated with the nearest cluster while keeping centroids as small as possible. This technique is extensively used in data cluster analysis and feature learning. The performance of the k-mean algorithm may vary because a slight change in data may lead to high variance. Therefore, it is rarely used in fall prevention or detection algorithms.

### 3.6. Linear Discriminant Analysis (LDA)

LDA is a supervised classification technique used for dimensionality reduction [78]. It is mainly used as a pre-processing step. The main goal of LDA is to transform the high-dimensional data into low dimensional data, which helps in reducing the cost and resources [79]. Usually, gait analysis acquires large data with similar patterns using wearables. Therefore, LDA helps reduce data dimensionality, especially when the processing is done at low-power devices.

### 3.7. Naive Bayes

Naive Bayes is also a supervised learning algorithm that works on the principle of the Bayes Theorem [80]. It is one of the simple and most widely used classification algorithms that can provide fast predictive results [81]. In general, it constructs classes based on probability using the Bayes theorem. Based on classes, irregular gait and fall can easily be detected quickly.

The algorithms mentioned above are primarily used in fall detection and prevention applications. In addition, there exist other algorithms for such applications, such as Logistic Regression, Dynamic Time Wrapping (DTW), and decision trees. Another way for identifying falls is to consider them as an anomaly detection problem. In such systems, autoencoders are used for detecting falls [82]. Autoencoder learns features by training the ADL models. Hence, fall activities are identified as an anomaly based on the reconstruction error. It consists of an encoder, decoder, and code layer. An encoder learns and compresses the important features of the input. The code layer acts as the middle layer that contains compressed and relevant data information. In comparison, the decoder transforms the data into original input. This method can help to reduce data dimensionality, obtained desired gait features, and detect unseen falls [83].

## 4. Literature Review

**Figure 6:**
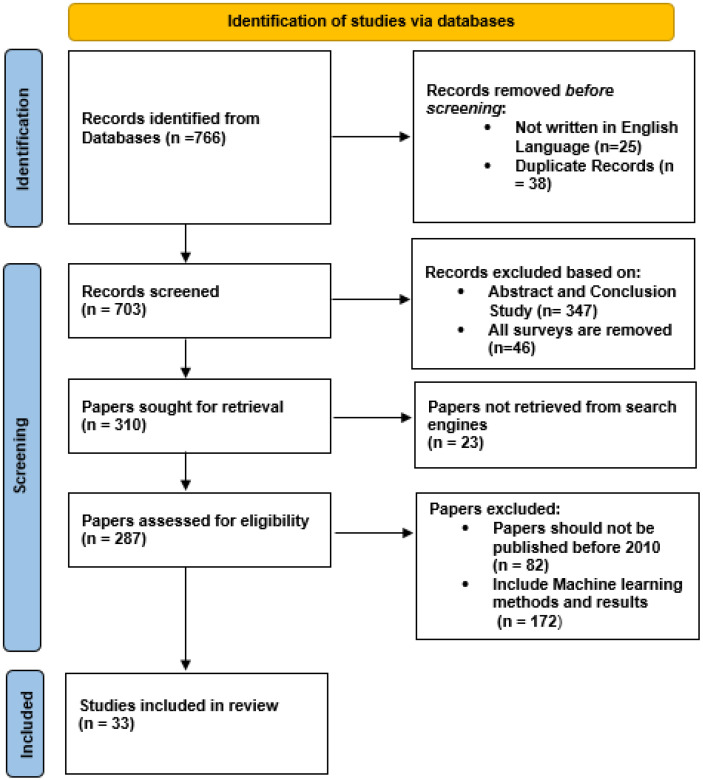
Proposed Methodology.

This review uses the Preferred Reporting Items for Systematic Reviews and Meta-Analyses (PRISMA) [84] technique for the paper selection, as presented in Figure 6. The PRISMA approach uses three steps for the paper selection:

1. Identification
2. Screening
3. Inclusion

The initial screening process provides approximately 16,890 results using the strings in Table 2. The results were more extensive using small strings such as “Fall Detection”, “Fall Prevention”, or “Machine Learning”. However, the use of multi-strings and long strings helps in reducing the dimensionality of the data. The screening process involves the identification of relevant publications using the title of the paper. It identifies 766 records, as shown in Figure 6.

The identification process involves checking for English language and duplicates and resulted in 703 publications for the screening process. The screening is a multiple-step process that analyses records based on abstract and conclusion. This helps in removing review papers. Furthermore, it performs the retrieval and eligibility checking. The eligibility check ensures that:

- The paper is published after 2010.
- The publishing venue is a Journal or Conference.
- The study is using ML for fall detection or prevention.
- The study is using a detailed methodology and results.

Through this scrutiny, we selected 33 papers for our review that were used for analysis. The overall analysis of selected papers is presented in the next section.

## 5. Analysis of Fall Detection and Prevention Schemes

We have shortlisted 33 papers related to fall detection or fall prevention. Each paper uses an ML algorithm and follows a particular methodology for data collection, feature extraction, and decision making. Twenty-nine papers describe fall detection methods in our analysis. In contrast, only four papers present fall prevention techniques using ML. The overall analysis is given below.

### 5.1. Data Collection

**Figure 7:**
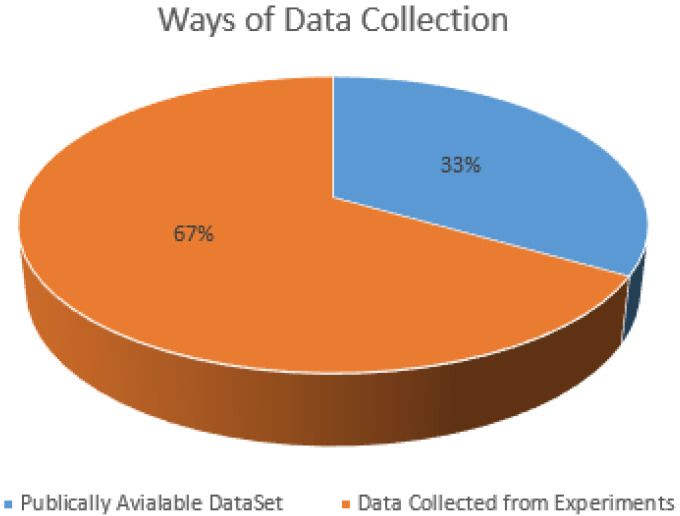
Methods of Data Collection.

Data collection is an essential part of fall detection or prevention systems. The available dataset is crucial for the working/results of the ML algorithms. The extensive and accurate dataset improves the accuracy of ML schemes for a specified task. Our analysis shows that the majority of the studies collected data in controlled lab conditions under supervision. The participants were trained to perform the falls and ADL for data acquisition. After training, a volunteer/participant performs fall and non-fall activities for data collection. Roughly 67% studies generated their datasets using the controlled lab environment. In contrast, only 33% of studies relied on publicly available datasets for their experiments, as shown in Figure 7.

#### Participants Age

**Figure 8:**
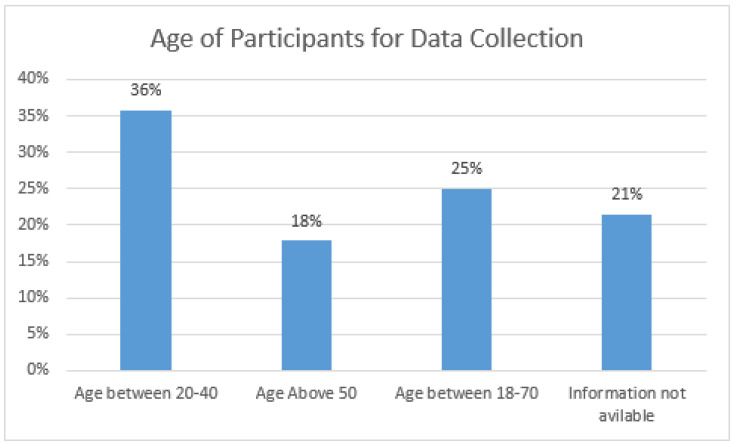
Age of Participants.

The majority of studies aim to develop a system for older people to detect and prevent falls. However, most volunteers in different studies are adults ranging between 20 and 40 years. Figure 8 illustrates that the percentage of this age group is 36%, double that of people aged above 50 years. Approximately 25% of the studies use the public dataset consisting of participants ranging between 18 and 70 years. Therefore, it is hard to classify them in a specific age bracket. Similarly, 21% of the studies did not reveal the age of volunteers.

### 5.2. Devices for Data Acquisition

**Figure 9:**
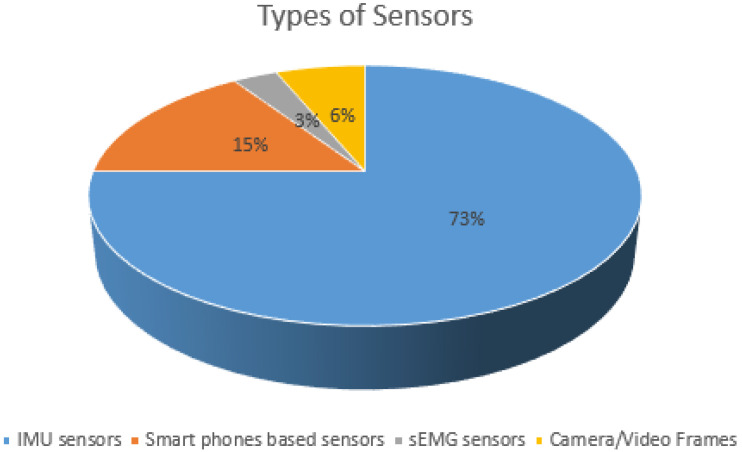
Types of Sensors.

The analysis shows that studies mainly used wearables for data acquisition. The frequently used wearables were mainly independent and phone-based IMUs. Figure 9 shows that the percentage of IMUs is 88%, including independent and phone-based IMUs However, IMUs consist of multiple sensors (accelerometer, gyroscope, and magnetometer). They can use single or multiple sensors to acquire the participant’s data. The analysis highlights that the accelerometer signal is used for the acquisition in 52% of the studies. In comparison, 30% of studies used gyroscope signals.

### 5.3. Sensors Placement

**Figure 10:**
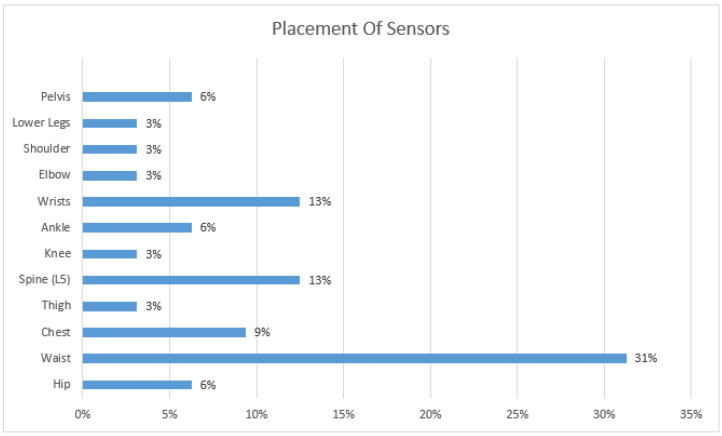
Placement of Sensors.

As explained earlier, the majority of studies use wearables. A wearable can be placed at multiple spots on a human body to measure different values, depending on the application requirements. Generally, a sensor should be placed so that the maximum movements and signals can be captured. Figure 10 shows different positions where sensors are placed for collecting data. The analysis shows that the frequently used locations are the waist, wrists, and spine.

### 5.4. Number of Sensors

**Figure 11:**
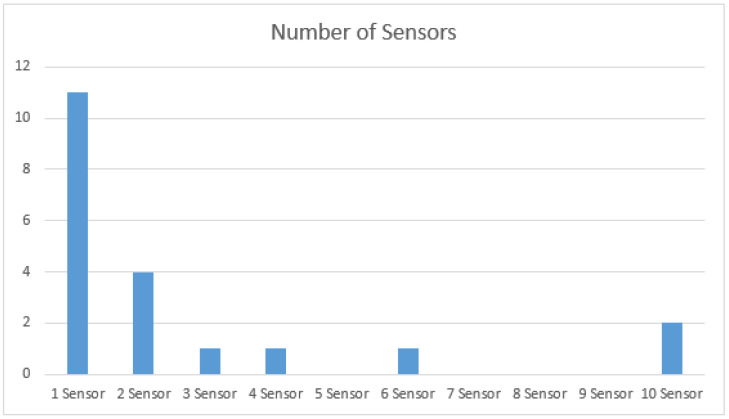
Number of Sensors.

Another critical factor for collecting data is the number of sensors involved in the collection process. Figure 11 illustrates that most studies rely on a single sensor’s data. However, some studies use multiple sensors such as 2,4 and even 10, as shown in the figure.

### 5.5. ML Algorithms

**Figure 12:**
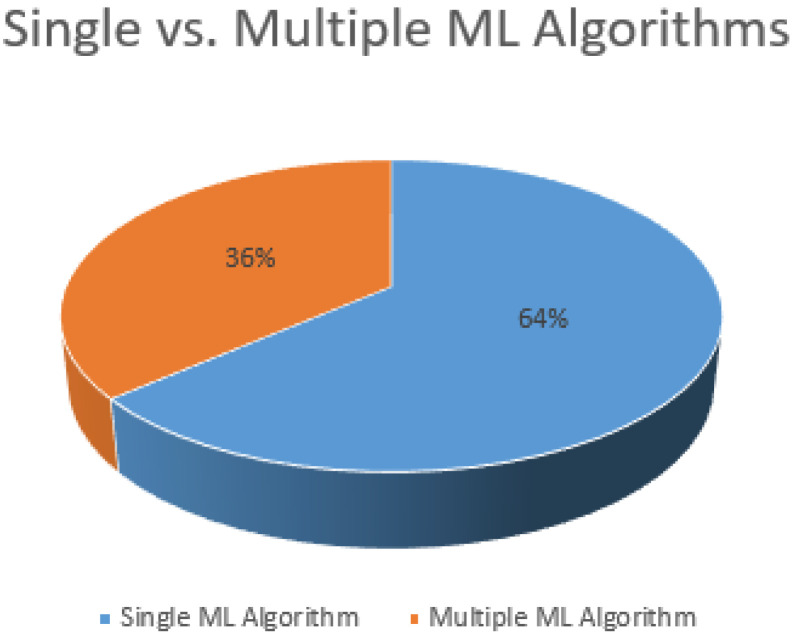
Use of Multiple or single machine learning algorithms.

**Figure 13:**
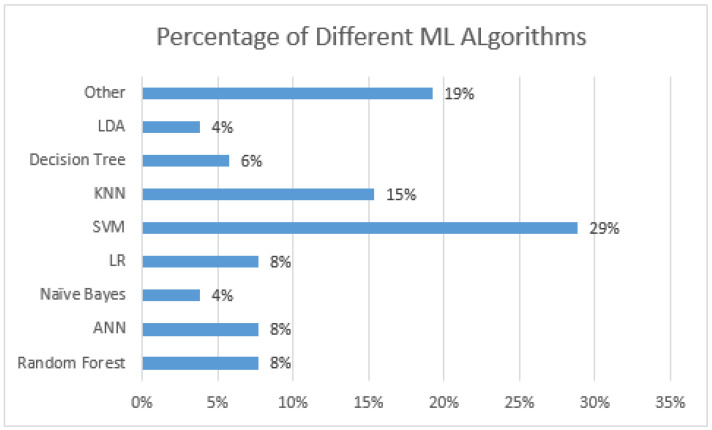
Frequency of Different Machine Learning Algorithms.

The most crucial factor of our paper is ML algorithms for performing the experiment and finding results. Depending on the methodology, authors used single as well as multiple ML algorithms. In the case of multiple algorithms, authors compared multiple algorithms for a specific task, such as fall detection, to identify the best-performing algorithm. Figure 12 shows that 36% of studies used and compared multiple ML algorithms. They includes a variety of supervised and unsupervised ML algorithms. Figure 13 lists the frequently used ML algorithms. It shows that the SVM is the most common choice for such systems, followed by kNN.

### 5.6. Performance Analysis

Analysis shows that different metrics are used for performance evaluation in different studies. However, it is difficult to compare all these studies using a specific evaluation metric such as accuracy. The primary reason for this is using the different datasets, acquisition processes, participants, environment, acquisition devices, device locations, and ML algorithm in different studies. However, for analysis, we present Table 3 showing the relationship between the ML algorithm, acquisition device, device location, and evaluation metrics.

For this paper, only the top four frequently used algorithms are considered to illustrate the findings. SVM using the IMU on the waist is the commonly used method for such systems. It also provides an average accuracy of 98% and 100% specificity and sensitivity. Therefore, this combination is a good starting point to design such fall detection systems. The results also indicate that independent IMU devices such as Shimmer’s IMUs provide better results than the mobile-based IMUs using any ML algorithm. The table highlights that RF’s performance is significantly low compared to SVM, KNN, and ANN. Table 4 shows the learning outcomes for ML algorithms and sensor placement deduced by the overall analysis.

An overview of all the selected papers is given in Table 5, and the discussion of all the findings is presented in Section 6.

## 6. Discussion

The previous section provides an overview and analysis of the recent studies. This section has a detailed discussion of the overall analysis. In the finings, it was observed that a majority of ML-based studies targetted fall detection only. The analysis shows that only 12% of studies used ML algorithms for fall prevention. Contrarily, a very high percentage (88%) of studies worked on fall detection. Having said that, the considered systems are not applicable in real life. For example, [54] uses a vision system in a controlled environment to observe the balance of participants. This system is not optimal for general usage. Similarly, the demographic characteristic and clinical data are used to prevent falls in [45]. This may help predict people with a high fall risk, but it is unable to identify the exact fall. Furthermore, the testing data for fallers in a controlled environment cannot guarantee high accuracy. One suitable solution to fall-prevention systems is to stimulate the muscle at the time of falling. Generally, fall prevention systems require very low latency (a few ms) to observe the irregularities in gait and prevent falls with muscle stimulation. Generally, it is hard for an ML algorithm to analyze and respond to situations in such a short period of of time. Furthermore, the muscle stimulation process is painful, and even low frequencies of false alarms make this system less suitable for usage. Therefore, we observe few fall prevention studies using ML. One more observation is the use of single or multiple ML algorithms. Approximately 36% of the papers use and compare multiple ML algorithms to find suitable detection or prevention algorithms. The use of multiple ML algorithms allows us to compare results based on the dataset, features extracted, the complexity of models. Hence, an optimal ML algorithm is selected for the desired application. The recent trends and analysis suggest that SVM is widely used over the years. The percentage of SVM usage is always high in the past ten years compared to the other ML algorithms. ANN and RF are the second and third most used algorithms over the years. One reason for SVM as the first choice of algorithm is its ability to identify distinct datasets. For example, the dataset/pattern of a fall and standing person can have a wide margin around the hyperplane. Furthermore, SVM does not suffer from overfitting and handles high-dimensional data effectively, which is the case with fall detection. One more advantage of SVM is its memory-efficient nature, which is optimal for wearables.

The data collection methods of the majority of the papers consisted of volunteers between 20 and 40 years. Almost 36% of the papers used volunteers between the ages of 20 and 40. In contrast, only 13% of papers invited older adults (above 50) for data acquisition. The main argument for choosing adults over the elderly is the physical dynamics. There are chances of hurting the old age group while performing the fall activities during data collection. However, the elderly group has a different and irregular gait from healthy adults, and the system is more relevant to such age groups. Therefore, it is necessary to acquire data from such groups. One of the observations is that the use of a controlled environment for system development and testing. Run time testing is not performed for the systems developed. For the realistic adoption of the system, it should ideally be tested in a real-time environment. Such systems can help to identify the issues or errors in real-time.

The performance of the ML algorithm depends on the features, dataset, and the proportion of training and testing data. Each study uses a different dataset, preprocessing algorithm, classifier, ML algorithm, acquisition device, and location that make it hard to compare based on specific parameters. Most systems are tested in a laboratory under a controlled environment that generates better results. However, if the same system is used for real-time testing, its accuracy and sensitivity may drastically change. For example, 100% accuracy was achieved by [93] when the system was tested in an offline environment. At the same time, the accuracy was 86% when tested on real-world data.

One last observation is that wearables are widely used in fall detection and prevention systems. The intuition is to develop a system with real-life applicability. Non-wearable systems are expensive and less scalable. Therefore, such systems can be restricted to certain places such as bedrooms and offices. However, people often perform such ADLs outside these places. Non-wearable systems often include cameras/video capturing that can easily compromise the user’s privacy. For these reasons, non-wearables were only used in 6% of studies. Users can wear wearables due to their portability (lightweight and size), which helps them to perform ADL in more realistic environments.

IMUs are the most frequently used wearables for fall detection and prevention. They consist of an accelerometer, gyroscope, and magnetometer. As a result, they provides 2 to 6 degrees of freedom, referring to different object movements in three-dimensional space. Depending on the experimentation and system design, such sensors can easily be attached to any part of the body, i.e., wrist, hip, and waist. The analysis shows that the waist is the most widely used location to attach such sensors. One reason is that the center of gravity (CoG) is located near the waist and can provide better measurements for gait or fall. Presently, the majority of smartphones have embedded IMUs. It allows the use of single or multiple sensors at a time. The data of multiple sensors (sensor fusion) reduces the uncertainty in the data. However, we rarely see the use of sensor fusion in the selected studies. Based on the detailed analysis and observations, this paper identifies the following future directions.

1. **Energy Efficiency:** A wearable-based system can be used in a more realistic environment. However, these sensors are tiny with limited lifetime and processing power. Therefore, energy efficiency algorithms [107,108] are required to improve the feasibility of such a system [109]. The use of an energy harvester can be another potential solution to enhance the significance of the system. Fog or edge computing [110,111] is also an exciting solution to mitigate the impact of resource-hungry ML algorithms. The processing at the edge can eliminate the computational load at the sensors. Therefore, it is optimal for designing a fall detection application. In contrast, edge computing introduces the delays that make it unfit for fall prevention applications.
2. **Datasets:** Most studies created a dataset for their experiments. However, the datasets were mainly small and consisted of healthy subjects. Extensive datasets improve the classification accuracy. Therefore, it is essential to generate large datasets, primarily consisting of elderly data. More real datasets should be created, as current datasets includes samples from ages under 40, which are physically different from people over 60. The data fusion of custom data with public datasets can generate more accurate results. The Generative Adversarial Network (GAN) [112] is also an interesting choice to enhance the datasets.
3. **Context Awareness:** Context awareness is another exciting future direction. Usually, fall prevention applications rely on gait. However, the gait of an individual varies from surface to surface [113]. For example, the gait of a person would be different on the standard floor and sand. Therefore, there is a need for a context-aware system that incorporates this problem and minimizes false alarms.
4. **Sensor Fusion:** Sensor fusion works on the principle of combining the data from multiple sensors to make a decision [114]. It helps in reducing the uncertainties in the data. Therefore, sensor fusion can be a potential future direction for fall detection and prevention systems.
5. **Wearable Design:** Generally, users will be wearing the sensor-based solution for longer intervals [115]. Sometimes, a system consists of more than one sensor and electrodes. This makes the design of a user-friendly system an interesting future direction. During our analysis, this aspect was totally neglected, which questions the real-life applicability of the system.

## 7. Conclusions

Old age directly impacts the physical, cognitive, and sensory functionalities. As a result, older persons find it difficult to perform regular ADLs. Furthermore, these reduced functionalities increase the chances of falls, which can have fatal consequences. Therefore, the development of fall detection or prevention systems is desired to mitigate the fall impacts. This paper presents an overview of the latest fall detection and prevention systems using ML. It analyses the systems on various parameters such as participant’s age, dataset, ML algorithms, sensors, and the desired location of sensors for a specific task. The analysis shows that SVMs and wearables are frequently used for fall detection and prevention applications. However, most studies are performed in a controlled environment with adults, limiting the applicability of these studies. This paper also visualizes the learning outcomes of ML algorithms, their usage, and performance matrix with different wearables. In the end, it lists vital future directions such as energy efficiency, sensor fusion, context awareness, and wearable design.

## Figures and Tables

**Table 1:** Overview of Wearable Sensor.

Name of Sensor	Functionality
Accelerometer	Measures the rate of change of velocity (acceleration) of an object along its axis.
Gyroscope	Measures rotational changes concerning orientation. Hence, it calculates the angular velocity along three axes, pitch (x-axis), roll (y-axis), and yaw (z-axis)
Magnetometer	Measures the relative change of a magnetic field, its direction, and strength.
Inertial Measurement Unit (IMU)	Consists of an accelerometer, gyroscope, and magnetometer. It provides 2 to 6 degrees of freedom, which refers to different object movements in 3-dimensional space.
Surface Electromyography (sEMG)	It is used for detecting potentials using electrodes placed on the skin using electrochemical transducer [42].

**Table 2:** Strings Used in Search Engines.

Academic Library	Search String
Google Scholar	⇒ Fall detection using machine learning ⇒ Fall prevention using machine learning ⇒ Fall classification using machine learning ⇒ Machine learning for Fall Classification ⇒ Fall Detection and Prevention Using Machine Learning ⇒ Detecting Fall in Elderly Using Machine Learning
IEEE Xplore	⇒ (((“All Metadata”:Fall Detection) AND “All Metadata”:(Machine Learning) //Filters Applied: 2010–2020 ⇒ (((“All Metadata”:Fall Prevention) AND “All Metadata”:Machine Learning) //Filters Applied: 2010–2020 ⇒ (((“All Metadata”:Fall Detection and Prevention) AND “All Metadata”:Machine Learning) //Filters Applied: 2010–2020
Science Direct	⇒Fall Detection Using Machine Learning. Limited to research articles, conference abstracts. ⇒Fall Prevention Using Machine Learning Limited to research articles, conference abstracts. ⇒Fall Classification Using Machine Learning Limited to research articles, conference abstracts.

**Table 3:** Performance Analysis of ML Algorithms using Different Sensors and Locations.

ML Algo	Sensor	Sensor Placement	Accuracy	Sensitivity	Specificity	References
SVM	IMU	Waist	98%	100%	100%	[46,52,53,85,86,87,88]
		Wrist	91.13%	99%	NA	[50,89]
	SmartPhone (IMU)	Waist	97.80%	99.50%	95.20%	[39]
		Thigh	91.70%	95.80%	88.00%	[39]
kNN	IMU	Wrist	99%	99%	NA	[89,90]
		Waist	99.78%	100%	99.91%	[89]
		5th lumbar vertebra and sacrum	99.40%	NA	NA	[91]
ANN	IMU	Waist	95.25%	96.50%	94.00%	[46,92]
	SmartPhone (IMU)	Wrist	92.96%	99.45%	100%	[93]
	IMU	L5 vertebra	96.3%	NA	NA	[94]
RF	IMU	Hip	73.70%	84%	NA	[95]
		Lower legs, posterior pelvis	77.30%	66.10%	84.70%	[96]
	SmartPhone (IMU)	Hand	NA	99%	98%	[51]

**Table 4:** Learning Outcomes from Analysis of ML Algorithms and Sensor Placement.

Learning Outcomes Using ML Algorithms	
**ML Algorithm**	**Learning Outcomes**
SVM	- The pattern of a falling and standing person can have a wide margin around the hyperplane. It can easily distinguish fallers from non-fallers. - SVM does not suffer from overfitting and handles high-dimensional data effectively; that is the case of fall detection. - It is memory-efficient in nature that is optimal for wearables.
kNN	- It is faster and learns from the datasets at time of making real time predictions. Therefore, small change in input data have not much effect on the classification results. - As classifier adopts to new data points, it can be used for classifying fall detection and prevention in real time. - The systems may be computationally expensive because a lot of memory is required to store the training data.
ANN	- Neural networks have the ability to learn by themselves and not depend only on the input data. After learning from the initial inputs and their relationships, ANNs can infer results from unseen data, making the model generalized. Therefore, when we provide unseen input data, the network learns the fall and non-fall activities and predicts better results. - As neural networks learn from examples, it can be helpful in determining falls in real-time conditions when input data may differ from training data.
RF	- The benefit of RF is that it is fast and effective with larger data. Therefore, it is a good choice to observe the fall activities or irregular gait for fall prevention. - The results of RF change considerably with minor changes in data due to large tree structures. The human gait is very dynamic and tends to change abruptly. Therefore, it often results in low accuracy and precision, as shown in the studies. - It consumes high memory, which makes it less efficient for wearable devices.
**Sensor Placement**	**Learning Outcomes**
Waist	- As the center of gravity lies around the waist, body movements can be measured accurately. Use of an accelerometer enables easily detecting the linear movements of the body, while a gyroscope can identify the turns or movement around the axis. - Therefore, sensors placed on the waist can help in identifying gait irregularities and be useful for fall detection and prevention. - Generally, a belt or pouch is required to wear the sensor on the waist, which may not be optimal for daily usage.
Wrist	- Provides good wear time compliance. - Wrist accelerometers can detect multiple intensities of activities that can be helpful in fall classification. - However, sometimes, the movement of the wrist may cause false alarms for fall detection. - Sedentary behavior (walking or lying down etc.) can be estimated accurately by a wrist-worn accelerometer. - Wrist-based sensors cannot predict lower body movements, so they are not suitable for fall prevention systems requiring minor gait details.
Hip	- Hip-worn sensors are limited in collecting data for different body movements. - A hip angle is similar for different walking activities, which makes it not feasible for fall prevention. - In contrast, hip angle/sensors can be useful in detecting falls.
Thigh	- Sensors worn on thigh can detect specific gait angles, making them useful for fall detection and prevention applications.

**Table 5:** Comprehensive Analysis of Selected Papers for Fall Detection and Prevention.

Ref.	Year	ML Algorithm(s)	Sensor	Placement of Sensors	Target Area	Tool(s)
Salleh et al. [46]	2020	Nonlinear Auto Regression neural network (NARnet)	Invensense sensor	Waist	Fall Detection	NA
Kumar et al. [47]	2019	SVM	IMU	Hip	Fall Prevention	DART
Chelli et al. [97]	2019	KNN, ANN, QSVM, and EBT IMU	NA	NA	Fall Detection	NA
Dubois et al. [54]	2019	Clustering	Microsoft Kinect v2 sensor (camera)	In front at 2 cm distance	Fall Prevention	NA
Kim et al. [44]	2019	Multi class Pre-Impact Fall Detection Model	IMU	Left anterior iliac crest of the pelvis	Fall Detection	NA
Wang et al. [45]	2019	Multi-view ensemble learning with missing values (MELMV)	NA	NA	Fall Prevention	NA
Santos at el. [98]	2019	Convolutional Neural Network	Smart phone or smart watch	NA	Fall Detection	NA
Villar et al. [99]	2019	SAX TS representation together with the TF-IDF	3DACC sensor	Wrist	Fall Detection	R Studio
Yacchirema et al. [100]	2019	DT, Ensemble, LR, Deepnets	IMU	NA	Fall Detection	NA
Hua et al. [95]	2018	Random Forest	IMU	Hip	Fall Detection	STEADI
Shahzad et al. [39]	2018	SVM	Mobile Phone	Waist, thigh	Fall Detection	MKL_SVM
Putra et al. [101]	2018	EvenT-ML	Shimmer sensors	Chest, Waist	Fall Detection	NA
de Quadros et al. [90]	2018	KNN, DT, SVM, LR, LDA	GY-80 IMU device	Wrist	Fall Detection	NA
Hussain et al. [86]	2019	DT, LR, KNN, SVM classifier with Quadratic kernel function.	IMU	Waist	Fall Detection	MATLAB
Hsieh et al. [87]	2018	SVM with RBF Kernel function	IMU	Waist	Fall Prevention	NA
Aicha et al. [92]	2018	Combines convolutional and recurrent models (ConvLSTM)	IMU	Lower Back	Fall risk	NA
Rescio et al. [102]	2018	LDA	sEMG	Lower limb (Gastrocnemius and Tibilias muscles)	Fall Detection	MATLAB
Rodrigues et al. [91]	2018	SVM, Boosted and bagged DT, kNN, k-mean, Hidden Markov models	IMU	5th lumbar vertebra and sacrum	Fall Detection	MATLAB
Saleh et al. [52]	2019	Two SVM combined	IMU	Waist	Fall Detection	NA
Serpen et al. [103]	2018	Random Forest, SVM	SHIMMER	Chest, thigh	Fall Detection,	NA
Hu et al. [29]	2018	Deep learning network (LSTM)	IMU	L5 vertebra	Fall Detection	Python
Mauldin et al. [93]	2018	NB, SVM, Deep Learning	Smart watch	Wrists	Fall Detection	NA
Drover et al. [96]	2017	Random Forest Classifier	IMU	Lower legs (left and right lateral shanks), posterior pelvis	Fall Detection	MATLAB
Fan et al. [104]	2017	Directed Acyclic Graph Support Vector Machine (DAGSVM)	NA	Video frames	Fall Detection	NA
Shawen et al. [51]	2017	Random forest, SVM, Gradient boosting, wXtreme Gradient Boosting (XGBoost)	Mobile Phones (Samsung Galaxy S4)	Waist, in a pocket, hand	Fall Detection	MATLAB and Python
Hsieh et al. [105]	2016	SVM, kNN	IMU	Wearable sensor	Fall Detection	NA
Bourke, et al. [9]	2016	DT	IMU	L5 (fifth lumbar spine)	Fall Detection	Matlab, Weka
Chen et al. [53]	2015	SVM	IMU	Waist	Fall Detection	NA
Özdemir et al. [89]	2014	kNN, LSM, SVM, Bayesian Decision Making (BDM), DTW, ANN	IMU	Head, chest, waist, right wrist, right thigh, and right ankle	Fall Detection	NA
Aziz et al. [85]	2014	SVM	Inertial sensor	Waist	Fall Detection	Matlab
Albert et al. [106]	2012	SVM, LR, Naïve Bayes, Class reg tree, KNN	Mobile phones	Attached on belt and centred at the back	Fall Detection	MATLAB
Caby et al. [50]	2010	A radial basis function network classifier, SVM, KNN, Naive bayesian	NA	Knee, ankle, wrists, elbow, shoulder (both left and right)	Fall Detection	NA
Shan et al. [88]	2010	SVM	IMU	Waist	Fall Detection	MATLAB

## Data Availability

Not applicable.

## Abbreviations

The following abbreviations are used in this manuscript:

**Acronym**	**Extended Meaning**
ADL	Activities of Daily Life
ANN	Artificial neural network
CoG	Center Of gravity
ConvLSTM	Combines convolutional and recurrent models
DAGSVM	Directed acyclic graph support vector machine
DT	Decision tree
DTW	Dynamic time warping
GRF	Ground reaction force
GAN	Generative adversarial network
IMU	Inertial measurement unit
ILFS	Infinite latent feature selection
KNN	k-nearest neighbor
LDA	Linear discriminant analysis
LR	Logistic regression
LRS	Laser range scanners
LSM	Least squares method
LSTM	Long short-term memory
ML	Machine learning
MELMV	Multi-view ensemble learning with missing values
MLP	Multi-layer perceptron
PRISMA	Preferred Reporting Items for Systematic Reviews and Meta-Analyses
QoL	Quality of life
SVM	Support vector machine
sEMG	Surface electromyography
XGBoost	Extreme gradient boosting
WHO	World health organization
